# Buschke-Löwenstein Tumor of the Vulva: Clinical Neglect, Molecular Continuum, and Pathogenetic Insights

**DOI:** 10.7759/cureus.106181

**Published:** 2026-03-31

**Authors:** Jesús Iván Martínez-Ortega, Frida Itzel Rosas-Lezama, Arely Gissell Ramirez Cibrian

**Affiliations:** 1 Histology, Autonomous University of Nuevo Leon, Monterrey, MEX; 2 Dermatology, Dermatology Institute of Jalisco, Zapopan, MEX; 3 Dermatology Service, General Zone Hospital 11, Mexican Institute of Social Security, Piedras Negras, MEX; 4 Internal Medicine, General Zone Hospital, Mexican Institute of Social Security, Merida, MEX; 5 General Medicine, Universidad Autónoma de Campeche, Campeche, MEX

**Keywords:** benign vulvar tumors, buschke-löwenstein tumor, epithelial hyperplasia, exophytic tumors, giant condyloma acuminatum, human papillomavirus (hpv), low-risk human papillomavirus (lr-hpv), neglected disease, surgical management, vulvar disease

## Abstract

Buschke-Löwenstein tumor (BLT), also known as giant condyloma acuminatum, is an uncommon manifestation of low-risk human papillomavirus (HPV) infection, characterized by massive exophytic growth and substantial local morbidity. Although histologically low-grade, BLT can become profoundly function-limiting and carries a variable risk of malignant transformation, particularly in long-standing or untreated lesions.

We report a case of a neglected giant vulvar BLT in an adult woman who presented after several years of progressive growth, resulting in impaired ambulation, hygiene difficulties, and sexual dysfunction. Surgical excision with margin control was performed, leading to functional improvement, and histopathological examination confirmed BLT, without evidence of invasive squamous cell carcinoma (SCC). This case highlights the clinical consequences of delayed diagnosis and limited access to care, positioning BLT as a paradigmatic clinically neglected disease. Emerging pathogenetic evidence supports the concept that BLT represents an extreme along a continuous spectrum of low-risk HPV-associated disease, in which sustained epithelial hyperplasia, stochastic persistence of infected basal cell clones, and local immune modulation permit progressive, space-occupying growth over time. Early recognition and definitive surgical management remain essential to prevent severe morbidity and reduce the risk of recurrence or malignant progression.

## Introduction

Buschke-Löwenstein tumor (BLT), also known as giant condyloma acuminatum, is an uncommon manifestation of anogenital human papillomavirus (HPV) infection, characterized by slow-growing, exophytic, verrucous masses with locally aggressive behavior. Although classically associated with low-risk HPV genotypes, particularly HPV 6 and 11, BLT exhibits a distinctive clinical course marked by progressive enlargement, functional impairment, and a propensity for local recurrence [1-3].

In contrast to conventional condyloma acuminatum, BLT lesions may reach massive dimensions over years, causing significant morbidity related to ambulation, hygiene, sexual function, and psychosocial well-being. Despite their alarming size and destructive appearance, these tumors are histologically low-grade in many cases and biologically distinct from verrucous carcinoma. This distinction has historically been blurred in the literature. This lack of terminological and diagnostic consensus has contributed to persistent misconceptions regarding the malignant potential and mortality of BLT [1-3].

Recent efforts have emphasized the importance of lesion size and extent in defining BLT. Proposed classification systems stratify tumors based on cumulative diameter, base width, circumferential involvement of the anogenital organ, and the presence of invasive carcinoma or infectious complications, underscoring that BLT represents a spectrum of disease rather than a uniform entity [1]. Within this framework, large and long-standing lesions pose increasing challenges in management and surveillance, particularly when diagnosis and treatment are delayed [1-3].

Vulvar BLT is especially rare and often underreported, and its presentation may be further complicated by social stigma, delayed healthcare access, and limited resources, leading to advanced disease at the time of evaluation [1-6]. Although approximately 200 cases have been reported in the literature, as noted in recent reviews [3], the true incidence is likely underestimated.

We report a case of a neglected giant vulvar BLT in an adult woman, highlighting its clinical presentation, diagnostic evaluation, histopathologic features, and surgical management, and discuss the implications of delayed diagnosis, functional burden, and contemporary concepts regarding classification and biological behavior.

## Case presentation

A 46-year-old woman presented with a long-standing history of a progressively enlarging vulvar mass, with an estimated duration of approximately four to six years. The lesion initially appeared as small verrucous papules and gradually coalesced into a massive exophytic tumor involving the vulvar region.

The patient reported a single lifetime sexual partner and denied prior sexually transmitted infections. Her partner was not evaluated. She had no relevant past medical or surgical history and denied immunosuppression. There was no known family history of malignancy or similar lesions. Serologic testing for syphilis, hepatitis B, hepatitis C, and human immunodeficiency virus (HIV) was negative. No prior diagnostic procedures or treatments had been performed before presentation.

**Figure 1:**
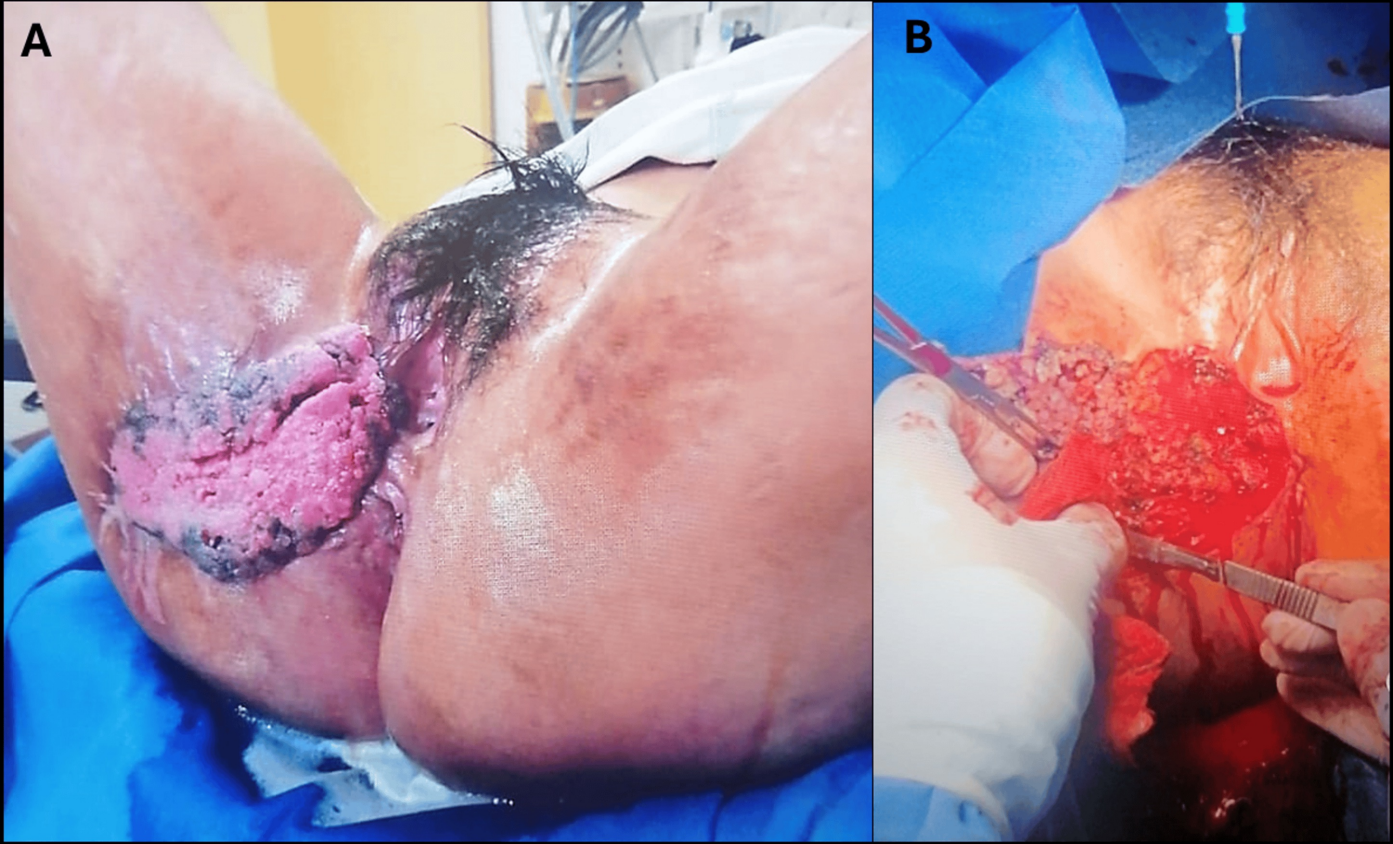
Buschke-Löwenstein tumor of the vulva: preoperative and intraoperative appearance (A) Preoperative clinical photograph showing a large, exophytic, verrucous, cerebriform vulvar mass with a macerated surface and irregular borders, causing marked distortion of the vulvar anatomy. (B) Intraoperative view during surgical excision, demonstrating the bulky, papillomatous architecture of the lesion and its predominantly exophytic growth pattern, without obvious deep infiltration.

On physical examination, a giant, cerebriform, multilobulated verrucous mass was observed, predominantly affecting the right labium majus and extending toward the perineal area. The lesion measured approximately 25 cm in its greatest horizontal diameter and 17 cm in the vertical axis. Areas of maceration, superficial erosion, and intermittent bleeding were noted, along with a malodorous discharge. The mass caused significant functional impairment, interfering with ambulation, personal hygiene, and daily activities. No clinically evident inguinal lymphadenopathy was detected (Figure 1A).

**Figure 2:**
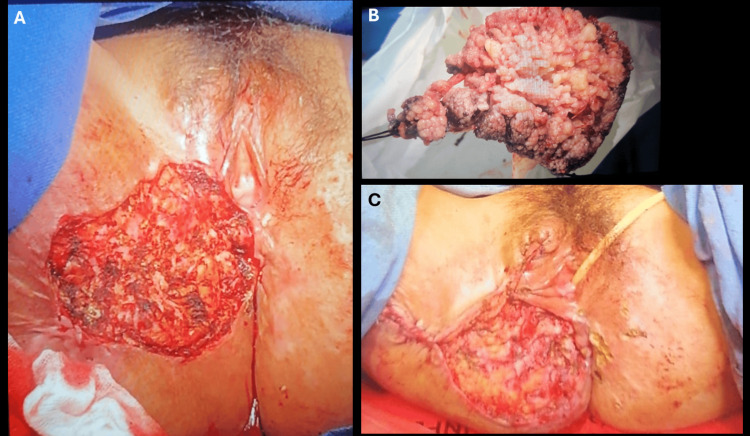
Surgical excision and postoperative appearance of vulvar Buschke-Löwenstein tumor (A) Intraoperative photograph of the resected specimen, revealing a multilobulated, cauliflower-like mass consistent with giant condyloma acuminatum (Buschke-Löwenstein tumor). (B) Gross specimen after complete excision, showing the characteristic exophytic and verrucous morphology. (C) Immediate postoperative view, demonstrating the surgical defect following wide local excision, managed with partial closure and secondary-intention healing.

Based on the lesion’s size, chronicity, verrucous morphology, and locally destructive behavior, a clinical diagnosis of giant condyloma acuminatum (BLT) was favored. The differential diagnosis included verrucous carcinoma and invasive squamous cell carcinoma (SCC). The patient underwent wide local excision with surgical debulking. Partial closure of the defect was performed, with placement of a Penrose drain, and healing by secondary intention (Figure 1B and Figures 2A-2C).

Histopathological examination demonstrated a papillomatous and verrucous squamous proliferation, characterized by exophytic growth with fibrovascular cores, marked epithelial thickening (acanthosis), and hyperkeratosis. Broad, pushing epithelial borders were observed, without evidence of stromal invasion. HPV genotyping detected HPV types 6 and 16.

**Figure 3:**
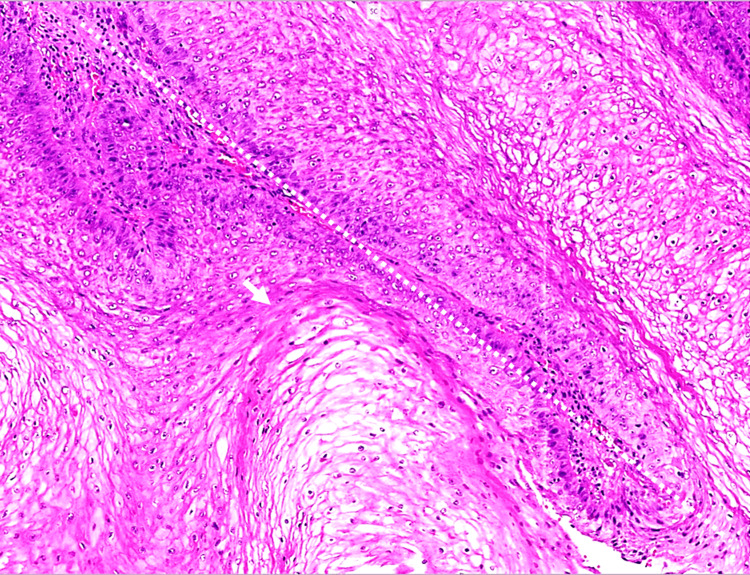
Histopathological features of vulvar Buschke-Löwenstein tumor Hematoxylin- and eosin-stained section (×20), showing papillomatous architecture (white arrow) and a fibrovascular core containing erythrocytes (dashed line), consistent with Buschke-Löwenstein tumor.

In the appropriate clinical context, the integration of histopathological features, HPV genotyping, and the characteristic exophytic growth pattern supports the diagnosis of giant condyloma acuminatum (BLT). No features of invasive SCC were identified (Figure 3), effectively excluding invasive SCC and verrucous carcinoma.

The postoperative course was uneventful. The Penrose drain was removed during the early postoperative period, and wound healing progressed by secondary intention without signs of infection. The patient reported improvement in local symptoms and mobility. Unfortunately, long-term follow-up was not available, as the patient was lost to follow-up due to socioeconomic constraints.

## Discussion

This case exemplifies the classical clinical behavior of BLT: a slowly progressive, exophytic, cerebriform mass that becomes profoundly function-limiting as it enlarges. In vulvar disease, this burden frequently manifests as impaired ambulation, hygiene difficulties, chronic maceration, and sexual dysfunction, often compounded by social stigma and delayed access to healthcare. In this sense, BLT represents a clinical form of neglect, driven by structural, social, and healthcare barriers that delay effective intervention. Parallels may be drawn with other HPV-related diseases, such as cervical cancer, that have been framed within global health literature as “neglected,” despite well-characterized biology and the existence of effective preventive strategies [3,4].

Although BLT is biologically and clinically distinct from verrucous carcinoma, exclusion of invasive SCC requires careful histopathological evaluation. Contemporary series demonstrate that invasion is not universal and correlates strongly with lesion size and chronicity, rather than with BLT per se. In a multi-institutional cohort of 38 BLT cases, invasive SCC was identified in approximately 50% of patients, while other large series report substantially lower rates [5,6]. Importantly, several studies describing “giant anal condylomas” or large anogenital verrucous lesions likely include biologically heterogeneous entities within the same category, further complicating interpretation. Collectively, these data support a nuanced oncologic framework: BLT is not uniformly malignant, but prolonged, unchecked growth increases the probability of malignant transformation, justifying timely surgical management and structured long-term surveillance [3,5,6].

The reported mortality of BLT has likewise been overstated in earlier literature. Initial reviews cited mortality rates of 20%-30%, figures that have been repeatedly propagated. However, detailed examination of these series reveals that most deaths were attributable to infectious complications, sepsis, or unrelated comorbid conditions, rather than direct tumor-related lethality. More recent cohorts with defined follow-up consistently demonstrate markedly lower disease-specific mortality, often below 3%, and, in several series, no fatalities attributable to BLT itself. These observations underscore the importance of distinguishing morbidity from mortality when counseling patients and framing prognosis [3,5,6].

From a histopathologic standpoint, both malignant transformation and mortality rates are further influenced by significant technical and selection biases. Complete histologic evaluation of massive, exophytic lesions is rarely feasible, and sampling is necessarily partial. This introduces an inherent risk of overestimating invasive carcinoma, based on focal findings that may not reflect the overall biological behavior of the lesion. In addition, historical conflation of BLT with verrucous carcinoma and the inclusion of smaller or frankly invasive tumors under the BLT designation have contributed to inflated malignancy statistics. As verrucous carcinoma is a well-differentiated variant of SCC that typically lacks HPV DNA, equating it with BLT obscures fundamental differences in pathogenesis and clinical trajectory [7].

The pathogenesis of BLT provides insight into its distinctive, massive, exophytic growth and helps reconcile its paradoxical behavior as a histologically low-grade, yet clinically aggressive, lesion. Unlike high-risk HPV, low-risk HPV types 6 and 11 encode E6 and E7 oncoproteins with quantitatively and qualitatively different effects on key tumor suppressor pathways [8-10]. E6 from HPV 6/11 induces p53 degradation in a cell density-dependent manner [11], while E7 preferentially targets p130, a regulator of cell-cycle exit in suprabasal epithelial layers. This molecular profile favors sustained proliferation in upper epithelial compartments, rather than early basal layer expansion, resulting in pronounced epithelial hyperplasia, papillomatosis, and expansive outward growth, rather than early invasion [8-10].

Emerging evidence suggests that clearance or persistence of HPV infection in immunocompetent hosts may depend not only on adaptive immune responses but also on stochastic proliferation dynamics of basal epithelial stem cells. Within this framework, BLT may represent a localized failure of clonal extinction, allowing long-term persistence of HPV-infected basal cell populations. Over time, sustained E6/E7 expression, particularly E7-mediated disruption of suprabasal cell-cycle control, may amplify epithelial hyperplasia and permit progressive, space-occupying growth over years [12].

Persistent E6/E7 activity may further contribute to a permissive local immune microenvironment. Experimental models indicate that E7-Rb family interactions, and associated epithelial hyperplasia, are linked to impaired antigen presentation, dysfunctional migratory dendritic cells, and ineffective local immune clearance, consistent with broader alterations in the HPV-associated immune microenvironment described in recent studies [13-18]. Taken together, these features support the concept that conventional condyloma acuminatum and BLT represent points along a continuous molecular and tissue-dynamic spectrum of low-risk HPV infection. While most lesions remain small and self-limited, rare cases progress to BLT when clonal persistence, epithelial compartment involvement, and local immune modulation converge. Secondary malignant progression appears to be driven primarily by lesion size, chronicity, and additional cofactors, such as smoking, immunosuppression, or high-risk HPV coinfection [3,6]. Clinicopathologic and molecular comparison between conventional condyloma acuminatum and BLT is presented in Table 1.

**Table 1:** Clinicopathologic and molecular comparison between conventional condyloma acuminatum and Buschke-Löwenstein tumor Comparative clinicopathologic and molecular features of conventional condyloma acuminatum and Buschke-Löwenstein tumor, supporting a spectrum model of low-risk human papillomavirus (HPV) infection. Data are summarized from published literature [9,10].

Feature	Conventional anogenital condyloma	Buschke-Löwenstein tumor (BLT)
Typical size	Small (<1-2 cm)	Giant (>5-10 cm), often >1/3 of organ circumference
Growth pattern	Limited, self-limited or slowly progressive	Massive, exophytic, cerebriform, relentless growth
Duration	Months	Years to decades
HPV genotype	HPV 6/11	HPV 6/11 (± high-risk coinfection)
Basal stem cell dynamics	Stochastic clonal extinction likely	Failure of stochastic clonal extinction
E6 activity	Weak, density-dependent p53 degradation	Persistent p53 modulation over time
E7 target preference	p130 > pRb	Sustained p130/pRb-family interference
Epithelial compartment affected	Predominantly suprabasal	Marked suprabasal hyperplasia with secondary basal persistence
Immune microenvironment	Effective local clearance	Locally permissive, impaired antigen presentation
Histology	Benign papillomatosis	Benign architecture with locally destructive growth
Risk of invasion	Minimal	Size- and chronicity-dependent, non-zero
Clinical framing	Common benign sexually transmitted infection (STI)	Neglected, morbid, locally aggressive disease

Management of vulvar BLT remains primarily surgical. Complete excision with margin control is the cornerstone of therapy, using wide local excision or staged debulking, depending on the extent and anatomical constraints. Reconstruction should prioritize restoration of function and wound healing, often necessitating partial closure and secondary-intention healing in extensive defects. Adjunctive therapies may be considered for residual disease, but are inferior as monotherapy due to high recurrence rates. Given the non-negligible risk of recurrence and delayed malignant transformation, long-term clinical surveillance is essential [3].

Interestingly, although current HPV vaccines are designed as prophylactic interventions, emerging clinical and immunological data suggest that vaccination may also influence viral persistence in individuals with established HPV infection. Observational studies in HPV-positive patients not previously vaccinated have demonstrated significantly higher rates of HPV DNA clearance following administration of the 9-valent vaccine, particularly when combined with local excisional treatment. While these findings do not establish a direct therapeutic effect on existing lesions, they support the concept that vaccine-induced immune activation can modify the natural history of persistent HPV infection [19].

In this context, BLT, characterized by massive antigen burden, prolonged clonal persistence, and relative genomic stability, may represent a biologically distinct scenario in which immune re-engagement could theoretically complement surgical management. Purpose-designed therapeutic HPV vaccines, including those targeting E7 and enhancing antigen presentation, have shown antitumor immune activity in preclinical models, and could represent a future adjunctive strategy [20,21].

A limitation of this report is the absence of long-term follow-up, as the patient was lost to follow-up due to socioeconomic constraints. This reflects a common challenge in patients with advanced BLT, and supports its characterization as a clinically neglected condition.

## Conclusions

BLT represents an uncommon but clinically significant extreme within the spectrum of low-risk HPV-associated anogenital disease. This case highlights how delayed diagnosis and limited access to care can permit indolent, histologically low-grade lesions to evolve into massive, function-limiting tumors with substantial morbidity. While BLT is not uniformly malignant, prolonged lesion persistence and increasing size elevate the risk of invasion, underscoring the importance of timely surgical management and long-term surveillance. From a pathogenetic perspective, BLT appears to arise from sustained epithelial hyperplasia driven by low-risk HPV E6/E7 activity, compounded by stochastic persistence of infected basal cell clones and local immune modulation.

Recognizing BLT as part of a continuous molecular and tissue-dynamic spectrum - rather than as a distinct malignant entity - helps reconcile its paradoxical biology and may improve diagnostic accuracy, prognostic counseling, and clinical management. Key learning points from this case include the importance of early recognition of rapidly enlarging verrucous lesions, the need for timely surgical intervention to prevent functional morbidity, and the value of integrating clinical, histopathological, and virological findings to support diagnosis. Greater awareness of BLT as a clinically neglected disease is essential to prevent advanced presentations and reduce associated morbidity.
